# Neurokinin-1 (NK-1) receptor and brain-derived neurotrophic factor (BDNF) gene expression is differentially modulated in the rat spinal dorsal horn and hippocampus during inflammatory pain

**DOI:** 10.1186/1744-8069-3-32

**Published:** 2007-10-31

**Authors:** Vanja Duric, Kenneth E McCarson

**Affiliations:** 1 Department of Pharmacology, Toxicology and Therapeutics, University of Kansas Medical Center, 3901 Rainbow Blvd., Kansas City, KS, 66160 USA

## Abstract

Persistent pain produces complex alterations in sensory pathways of the central nervous system (CNS) through activation of various nociceptive mechanisms. However, the effects of pain on higher brain centers, particularly the influence of the stressful component of pain on the limbic system, are poorly understood. Neurokinin-1 (NK-1) receptors and brain-derived neurotrophic factor (BDNF), known neuromediators of hyperalgesia and spinal central sensitization, have also been implicated in the plasticity and neurodegeneration occurring in the hippocampal formation during exposures to various stressors. Results of this study showed that injections of complete Freund's adjuvant (CFA) into the hind paw increased NK-1 receptor and BDNF mRNA levels in the ipsilateral dorsal horn, supporting an important role for these nociceptive mediators in the amplification of ascending pain signaling. An opposite effect was observed in the hippocampus, where CFA down-regulated NK-1 receptor and BDNF gene expression, phenomena previously observed in immobilization models of stress and depression. Western blot analyses demonstrated that in the spinal cord, CFA also increased levels of phosphorylated cAMP response element-binding protein (CREB), while in the hippocampus the activation of this transcription factor was significantly reduced, further suggesting that tissue specific transcription of either NK-1 or BDNF genes may be partially regulated by common intracellular transduction mechanisms mediated through activation of CREB. These findings suggest that persistent nociception induces differential regional regulation of NK-1 receptor and BDNF gene expression and CREB activation in the CNS, potentially reflecting varied roles of these neuromodulators in the spinal cord during persistent sensory activation vs. modulation of the higher brain structures such as the hippocampus.

## Introduction

To date, pain-induced peripheral and central sensory activation has been well characterized; however, little emphasis has been placed on studying the physiological mechanisms of the stress-like component of pain and its relationship to mood or affect. The importance of the emotional aspects of chronic pain and their impact on cognition and the overall perception of the nociceptive stimuli is augmented by clinical observations that majority of chronic pain patients often suffer from various forms of depressive illnesses [1-4]. The hippocampus, one of the main regulators of affect within the limbic system, has been previously shown to exhibit a robust stress-induced neurodegenerative plasticity related to the pathophysiology of depression [5-8]. Furthermore, the hippocampus has also been associated with the processing of pain-related information, particularly its potential role in shaping the affective-motivational response to noxious sensory stimulation. For example, peripheral administration of formalin was shown to attenuate levels of Fos protein in the rat hippocampus [9], while microinjections of lidocaine or glutamate receptor antagonists directly into the dorsal hippocampal formation decreased formalin-related nociceptive behaviors [10,11].

The tachykinin neuropeptide substance P (SP) and brain-derived neurotrophic factor (BDNF), each expressed by a subset of primary sensory neurons, are known modulators of nociceptive processing within the CNS [12-14]. Upon tissue injury or noxious stimulation, SP and BDNF are released into laminae I and II of the spinal cord dorsal horn, where through activation of their respective postsynaptic receptors, neurokinin-1 (NK-1) and tyrosine kinase B (trkB), contribute to development of hyperalgesia and central sensitization associated with chronic pain [15-18]. Both NK-1 receptors and BDNF are also highly expressed in the limbic system, primarily the amygdala, the hippocampus and the hypothalamus [14,19,20]. Their potential involvement in the processing of mood/affect has been suggested by clinical observations that NK-1 receptor antagonists have antidepressant properties [21,22], while amplification of hippocampal BDNF levels is considered to be a possible common down-stream effect of various antidepressant psychopharmacotherapies [23,24]. However, the influences of these neuromediators on modulation of neuronal plasticity following chronic pain, particularly their functional differences in the spinal dorsal horn vs. the hippocampus, are still largely undefined.

Previous studies have shown that intracellular transcriptional regulation of NK-1 receptor and BDNF genes, during either spinal nociceptive processing or stress-related stimulation of hippocampus, may be modulated by transduction pathways involving activation of extracellular signal-regulated kinases (ERK)/cAMP response element binding protein (CREB) cascades [25-29]. Once activated by wide variety of extracellular signals through dual phosphorylation at threonine (Thr^202^) and tyrosine (Tyr^204^) sites [30], p-ERK proteins translocate from the cytoplasm into the nucleus and activate transcription factors such as CREB. Subsequently, CREB phosphorylated at serine^133 ^(p-CREB) further induces transcription of genes containing cAMP response element (CRE) binding sites in their promoter regions [31], such as c-fos, NK-1, BDNF, and trkB [32-34]. The ERK/CREB-dependent cascade represents one of many intracellular pathways through which the extracellular stimuli, such as pain, may be transduced into post-translational and transcriptional responses within the neuronal tissue [25].

To address nociceptive regulation of regions of the CNS related to potentially distinct sensory vs. affective functions, we measured NK-1 receptor and BDNF gene expression in the spinal cord and the hippocampus following administration of complete Freund's adjuvant (CFA) into the rat hind paw. Furthermore, Western blot analysis was used to assess whether the changes in transcription of these two genes was correlated with CFA-evoked alterations in amounts of nuclear p-ERK and p-CREB proteins.

## Methods

### Animal housing and handling

Young adult male Sprague Dawley rats (Harlan Farms, Indianapolis, IN), used for all experiments, were age matched (7–8 weeks old) at the beginning of the treatments. All animals were allowed at least one week of habituation before any treatments were applied. The maintenance of the rat colony and all the animal treatments were in accordance with NIH laboratory care standards and approved by the University of Kansas Medical Center Institutional Animal Care and Use Committee. Efforts were made to minimize animal suffering and to reduce the number of animals used in this study. Rats were housed (12 h light/dark cycle) in groups of three per cage with *ad libitum *access to food and water; they were mixed together so there is one member of each treatment group in every cage. All rats, including the sham control group, were handled the same way to reduce the effects of stress associated with handling on the results.

### Experimental design

Rats (200–300 g) received a subcutaneous (s.c.) injection of 50 μL of Complete Freund's Adjuvant (CFA) (Sigma Chemical Co., St. Louis, MO) into the plantar aspect of the right hind paw. To establish a time-course of CFA's effects, rats were decapitated at several time points after CFA injection: 24 h (n = 5 or 6), 4 days (n = 6) and 10 days (n = 6). In order to address the long-term effects of peripheral nociception, two additional groups of animals received either a single (1×; CFA injection on day 0; n = 6) or triple (3×; CFA injections on days 0, 7 and 14; n = 5 or 6) CFA administrations over a period of 21 days. Sham control (n = 5 or 6) animals received no injection of CFA into the hind paw, but were momentarily restrained and their right hind paw manipulated. Otherwise, controls were handled identically to the treatment animals in terms of housing regime, daily transport from the animal facilities to the laboratory, and interactions with the handler.

### Tissue dissection

Immediately after decapitation, rat brains and spinal cords from the same animals were removed. Brains were dissected along the sagittal midline, followed by bilateral removal of the hippocampus. Spinal cord tissues were rapidly removed using hydraulic pressure (a forceful injection of ice-cold isotonic saline) applied to the caudal end of the vertebral canal with a 60 ml syringe and a 16-gauge needle. The lumbar portions (L1–L6) of the vertebral column were then dissected. The dorsal horn regions were dissected by cutting the lumbar portion of the spinal cord along the sagittal axis and dividing it into quarters. Only the ipsilateral side of the spinal cord was assayed.

### Solution hybridization – nuclease protection assays

The NK-1 receptor and BDNF (BDNF cDNA plasmid was graciously provided by Ronald Duman, Ph.D., Yale Medical Center) sense and antisense cRNA probes were generated by an *in vitro *run-off transcription reaction [23,35]. Synthesis of the antisense ^32^P-labeled cRNA probes using [α-^32^P]UTP (3000 Ci/mmol) was based on the protocol suggested by Promega (Madison, WI). Probes were purified through a NucAway spin column (Ambion, Austin, TX), and DNA template was subsequently digested using RQ1 Dnase (Promega, Madison, WI). The total cellular RNAs were extracted from both the hippocampal and spinal tissue samples using a rapid quanidinium isothiocyanate-phenol/chloroform extraction method and then assayed for NK1, BDNF or β-actin mRNAs using solution hybridization – nuclease protection assays as previously described [35-37]. Specific mRNA amounts were determined by comparison to cRNA quantitation standards. Levels of β-actin mRNA, unaffected by peripheral inflammatory nociception in either spinal cord or the hippocampus, served as gel loading controls and to ensure that the detected changes in NK-1 receptor and BDNF mRNA levels were not due to a CFA-related global modulation of gene expression within the CNS. Data values for NK-1 receptor and BDNF gene expression are reported as pg specific mRNA/ng β-actin mRNA [mean ± S.E.M.].

### Western blot analysis

Fresh ipsilateral dorsal horns of the spinal cord and bilateral hippocampal tissues were initially homogenized in lysis buffer containing a cocktail of phosphatase and proteinase inhibitors, followed by the isolation of the nuclear fractions using the Nuclear extract kit from Active Motif (Carlsbad, CA). Tissue protein concentrations were determined using the BCA protein assay kit (Pierce, Rockford, IL). Protein samples were electrophoretically separated on an SDS-PAGE gel (10% Tris-HCl; Bio-Rad, Hercules, CA) and transferred to polyvinylidene difluoride membranes (0.2 μm pores; Millipore, Bedford, MA). The membranes were blocked with 2% bovine serum albumin (BSA) for 2 h at room temperature (RT) and then incubated with overnight at 4°C with anti-p-ERK (detects ERK1/2 MAPKs phosphorylated at Tyr^204^; 1:200; Santa Cruz Biotechnology) or anti-p-CREB (detects CREB phosphorylated at Ser^133^; 1:1000; Upstate Cell Signaling Solutions) primary antibodies. All antibodies were diluted in Tris-buffered saline solution containing 0.5% Tween 20 (TBST) and 0.2% BSA. After washing in TBST, the blots were incubated with horseradish peroxidase (HRP)-conjugated secondary antibody for 2 h at RT (1:2000, Santa Cruz Biotechnology for p-ERK1/2; and 1:70000, Jackson Laboratories for p-CREB). Following the rinse in TBST, the blots were developed using enhanced chemilluminescence for 1 min and exposed onto Kodak MR autoradiographic film. To obtain loading controls, the blots were incubated in stripping buffer (1 M glycine pH 7.0, 20% SDS) for 1 h at 60°C and reprobed with antibodies recognizing total protein (1:1000, Cell Signaling Technology primary antibody for ERK1/2 or CREB; secondary antibody: 1:10000, Santa Cruz Biotechnology). Autoradiographs were scanned using a Gel-Doc imaging system (Bio-Rad, Milan, Italy) and analyzed with Quantity One 1-D software (Bio-Rad, Hercules, CA).

### Statistical analysis

Data from all the experiments were analyzed using analysis of variance (ANOVA) with either Fisher's PLSD or Student-Newman-Keuls' tests used for post-hoc comparisons. Significance was considered to be p ≤ 0.05.

## Results

### Nociception-evoked regulation of NK-1 receptor and BDNF gene expression in the spinal cord and hippocampus

**Figure 1:**
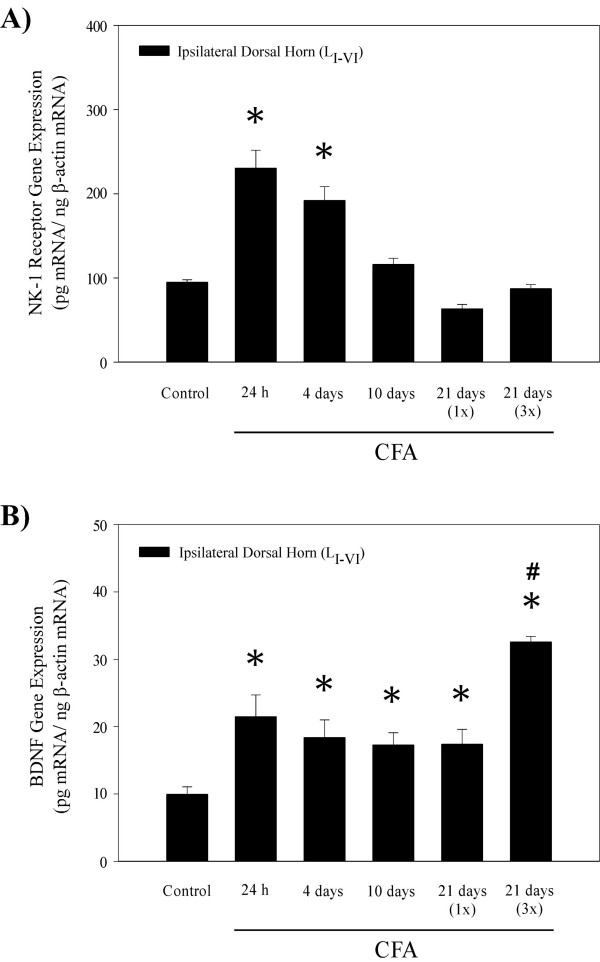
Histograms showing levels of NK-1 receptor and BDNF mRNA in the ipsilateral dorsal horn of the rat spinal cord 24 h, 4 days, 10 days and 21 days after a unilateral injection of complete Freund's adjuvant (s.c.) into the right hind paw. **A) **NK-1 receptor gene expression was significantly increased at 24 h and 4 days following CFA injection, with more robust response occurring at the earliest time point. At 10 and 21 days post CFA treatment NK-1 receptor mRNA levels were similar to sham controls. **B) **CFA treatments evoked increases in ipsilateral dorsal horn BDNF gene expression at all time points, with most robust up-regulation occurring at 21 days, following a triple CFA treatment. Data are expressed in pg mRNA/ng β-actin mRNA (Mean ± S.E.M.; n = 6); *p < 0.05 compared to the sham control group [ANOVA and Fisher's PLSD]; # < 0.05 compared to the CFA over 21 days (1×; single injection) [ANOVA and Fisher's PLSD].

As a model of persistent, peripheral inflammation, we used 50 μL injections of CFA into the plantar surface of the hind paw, which produced robust local swelling, erythema, and an overall hypersensitivity of the injected paw that lasted for days. Twenty-four hours after the subcutaneous injection of CFA into the right hind paw, NK-1 receptor mRNA levels were significantly increased by 144% in the ipsilateral dorsal horn (Fig. 1A), supporting our previous findings [35,38]. At 4 days post CFA treatment, NK-1 receptor gene expression was still up-regulated, but by day 10, NK-1 receptor mRNA levels were similar to sham controls. At 21 days, neither a single (1×) nor a triple (3×; injections on days 0, 7 and 14) CFA administration produced a significant increase of NK-1 receptor gene expression in the spinal cord. Figure 1B shows that nociceptive regulation of the BDNF gene initially appeared similar to NK-1, as CFA increased BDNF mRNA levels at 24 h and 4 days post-treatment. However, BDNF gene expression was continuously up-regulated throughout the entire time course, with most robust response occurring at 21 days after three weekly (3×) injections of CFA (228% increase in BDNF mRNA levels when compared to sham controls).

**Figure 2:**
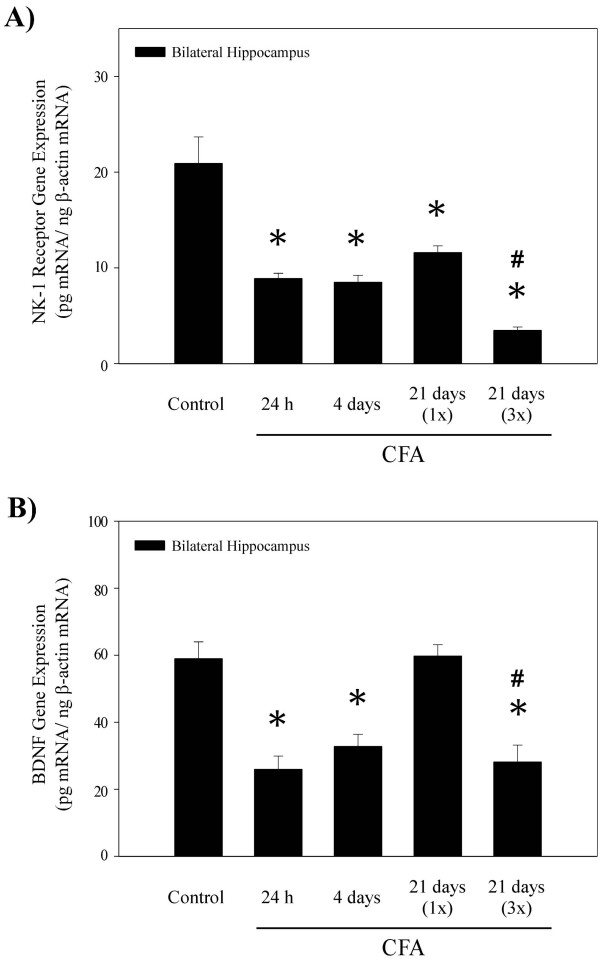
Histograms showing NK-1 receptor and BDNF mRNA levels in the rat hippocampus 24 h, 4 days, and 21 days following a unilateral injection of complete Freund's adjuvant (s.c.) into the right hind paw. **A) **Hippocampal NK-1 receptor gene expression was significantly decreased bilaterally at all time points post CFA injection. The most robust decreases in NK-1 receptor mRNA levels occurred at 21 days after a triple CFA injection; significant difference was observed when compared to either the control group or a single CFA injection 21 days group. **B) **CFA evoked bilateral decreases in hippocampal BDNF mRNA levels at 24 h and 4 days post injection. Note that at 21 days, a single CFA treatment (1×) had no effect, while a triple injection (3×) induced a significant down-regulation of BDNF gene expression. Data are expressed in pg mRNA/ng β-actin mRNA (Mean ± S.E.M.; n = 6); *p < 0.05 compared to the sham control group [ANOVA and Fisher's PLSD]; #p < 0.05 compared to the CFA over 21 days (1×; single injection) [ANOVA and Fisher's PLSD].

The effects of peripheral nociception on the hippocampus differed from those found in the spinal cord. Due to the unilateral nature of the pain treatment, all the gene expression assays were initially performed on both the ipsilateral and contralateral sides of the hippocampus. However, the mRNA levels are shown as hippocampal bilateral average, since CFA did not evoke any sided differences in expression of either NK-1 receptor or BDNF genes. Figure 2A demonstrates that injections of CFA significantly decreased hippocampal NK-1 receptor mRNA levels, similar to our previous findings after formalin administration [36]. Down-regulation of NK-1 receptor gene expression occurred as early as 24 h and persisted through the 21^st ^day post-CFA administration. Unlike the spinal cord, the largest changes were observed in rats that received triple CFA (3×) injections, which reduced NK-1 receptor mRNA levels by 83%. Likewise, hippocampal BDNF mRNA levels were also significantly reduced at 24 h and 4 days after CFA treatments (Fig. 2B). Interestingly, when measured at 21 days post CFA injection (1×), no changes in BDNF gene expression were observed, suggesting that the effects of a single CFA treatment on the hippocampal BDNF are less persistent than in the spinal cord. Moreover, three injections (3×) evoked similar diminishing effects on hippocampal BDNF mRNA levels as those observed at earlier time points.

### Nociception-evoked activation of ERK and CREB proteins in the spinal cord and hippocampus

**Figure 3:**
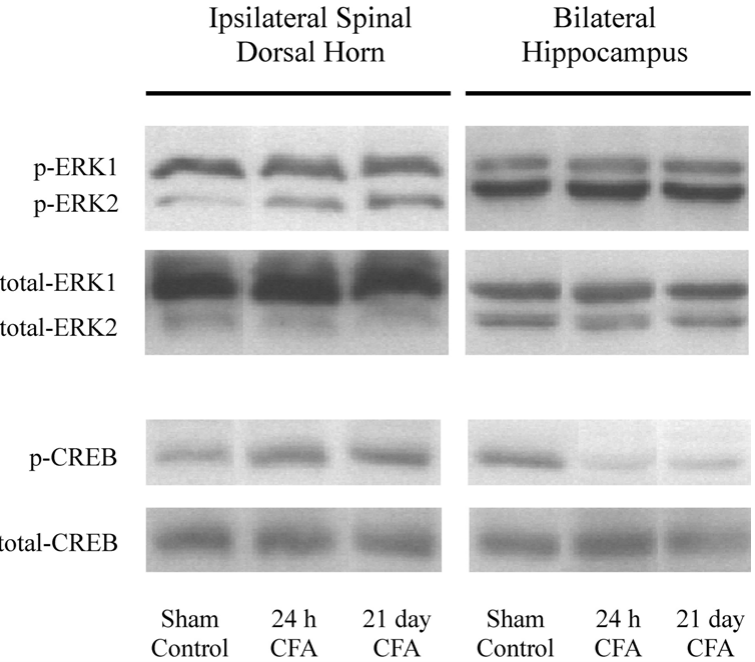
Representative images of Western blot analysis showing the effects of CFA-induced hyperalgesia on p-ERK1/2 and p-CREB protein levels in the rat hippocampus vs. the ipsilateral dorsal horn of the spinal cord. Total protein from nuclear fractions of the tissue was immunoblotted with either monoclonal anti-phosphoERK1/2 (p-ERK1: M_r _= 44 kDa; p-ERK2: M_r _= 42 kDa) or monoclonal anti-phosphoCREB (p-CREB: M_r _= 43 kDa) antibody. Tissue levels of constitutively expressed total ERK and total CREB proteins were used as loading controls. Proteins were visualized using secondary antibodies conjugated to HRP and a chemilluminescence detection system.

**Figure 4:**
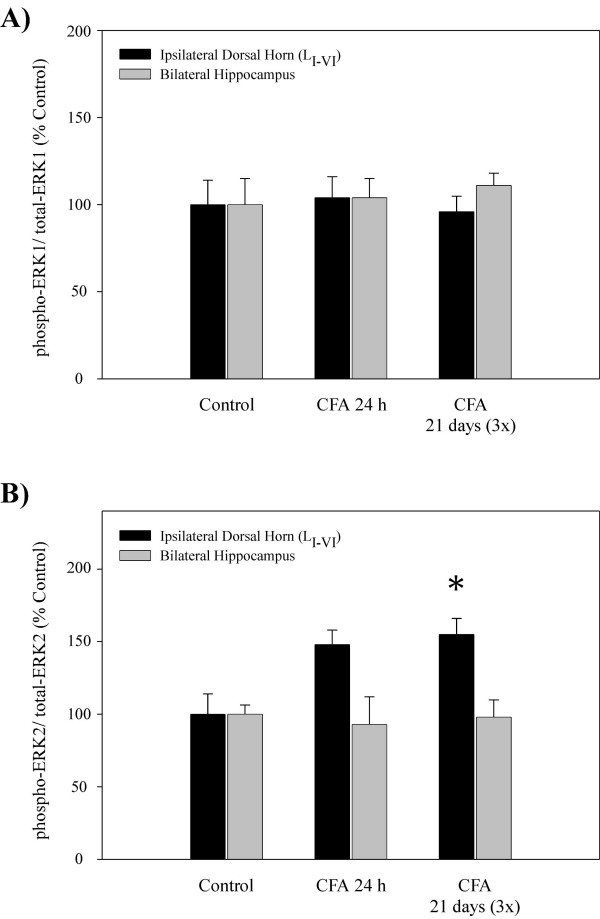
Histograms showing quantitative results of Western blot analysis for activation of ERK proteins 24 h (single injection) and 21 days (three injections) after a unilateral administration of complete Freund's adjuvant (s.c.) into the right hind paw. **A) **CFA had no effect no phosphorylation of ERK1 in either ipsilateral dorsal horn of the spinal cord or bilateral hippocampus. **B) **Hippocampal levels of p-ERK2 protein were not affected by the CFA treatment; however, in the dorsal horn CFA evoked a significant increase in p-ERK2 at both 24 h and 21 days after the administration. Optical density values are expressed as a ratio between the phosphorylated ERK (activated form) and total ERK (inactive form). Data are shown as % increase over sham control (n = 5); *p < 0.05 compared to control group (ANOVA and Student-Newman-Keuls' *post-hoc *test).

**Figure 5:**
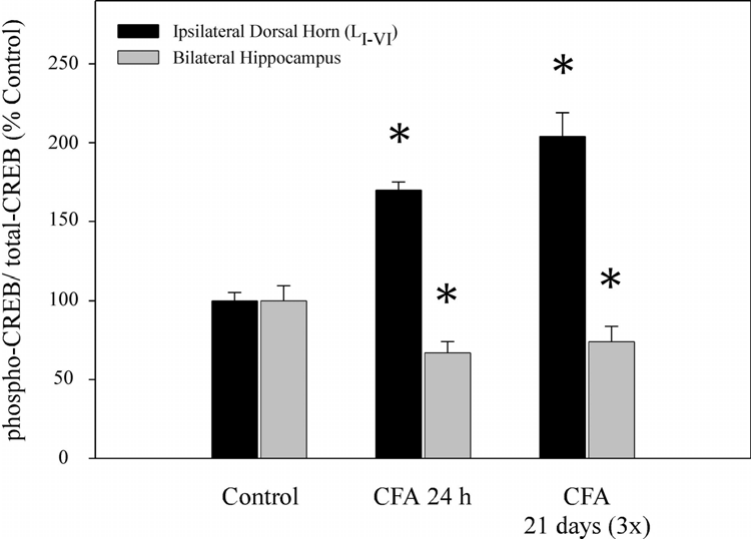
Quantitative results of Western blot analysis showing the effects of CFA-evoked peripheral nociception on activation of CREB protein. CFA increased phosphorylation of CREB in the ipsilateral dorsal horn at 24 h (single injection) and 21 days (three injections) post administration. Note that in the hippocampus the same treatment produced an opposite effect; p-CREB levels were significantly reduced at all time points. Optical density values are expressed as a ratio between the p-CREB (activated form) and total CREB (inactive form). Data are shown as % increase over sham control (n = 5); *p < 0.05 compared to control group (ANOVA and Student-Newman-Keuls' *post-hoc *test).

In order to address the intracellular signal transduction pathways that may underlie the regulation of NK-1 receptor and BDNF gene expression during inflammatory pain in the CNS, we measured changes in the activation of ERK1/2 and CREB proteins using Western blot analysis (Fig. 3). Tissue levels of total endogenous ERK and CREB proteins were also determined and used as SDS-PAGE loading controls; the data are expressed as increases in phosphorylated protein when compared to the total protein levels. Figure 4A shows that phosphorylation of ERK1 (p44) protein was not significantly altered in either the dorsal horn or the hippocampus at 24 h post-CFA treatment, nor after three weekly injections administered over 21 days. Similarly, CFA produced no apparent changes in activation of the hippocampal ERK2 (p42) either; however, in the spinal cord, a 55% increase in levels of p-ERK2 were observed 21 days after three CFA treatments (Fig. 4B). Phosphorylated CREB, which may be activated by various signaling pathways, can directly modulate the expression of NK-1 receptor and BDNF genes through the CRE sites on their promoter regions. As indicated on Figure 5, CFA increased levels of nuclear p-CREB protein in the ipsilateral dorsal horn at 24 h and 21 days (70% and 104% increases, respectively), while in the hippocampus phosphorylation of CREB was significantly diminished (a 33% decrease at 24 h vs. 26% decrease at 21 days), indicating a similar region-dependent pattern of regulation as observed with NK-1 receptor and BDNF genes.

## Discussion

The mechanisms by which inflammatory nociception is processed within the spinothalamic pathways of the CNS have been well established at both cellular and molecular levels. However, the impact of painful stimulation on the higher brain centers, mainly the emotion- and cognition-processing components of the CNS, is still unclear. Besides the sensory aspects of nociception, a negative impact on affect is essential for accurate characterization of a stimulus as "painful". Therefore, improved understanding of how pain affects the mood-controlling regions of the brain would be very beneficial to treatment of chronic pain in the clinical setting.

The experiments in this study were designed to conduct a direct comparison of CFA-induced peripheral nociception on the expression of NK-1 receptor and BDNF in the spinal cord and hippocampal formation, one of the central limbic regions involved in the regulation of affect. Current results demonstrate that NK-1 receptor and BDNF mRNA levels are initially increased in the ipsilateral dorsal horn at 24 h and 4 days post CFA (Fig. 1), consistent with results of other studies [35,39,40]. Previous reports have suggested that SP and BDNF may co-localize in the same large dense-core vesicles (LDCVs) of the small diameter primary nociceptors, and that their release is dependent on the activation of tyrosine kinase A (trkA) receptors due to enlarged levels of nerve growth factor (NGF) in the periphery [14]. Increases in NK-1 receptor and BDNF gene expression in the dorsal horn likely represent a secondary effect resulting from facilitated activation of NMDA, NK-1 and trkB receptors located on the secondary afferents. This effect is considered to be directly related to increased SP and BDNF release from the terminals of primary nociceptors in response to peripheral inflammation [15].

Another important aspect of this study was to address the long-term effects of nociception on CNS gene plasticity. Thus, 10 and 21 day time points were included in the experimental design. Previously we reported that a single injection of CFA produces thermal and mechanical hyperalgesia for about 10–12 days post injection; however, when CFA was administered three times (weekly injections) over 21 day period, hyperalgesia was present throughout the entire time course [41]. Therefore, we also compared the effects of single vs. triple CFA treatments on gene expression over a 21 day period. Spinal NK-1 receptor gene expression was not affected at these later time points (Fig. 1A), while the BDNF mRNA levels continued to increase, with the most robust response occurring after three weekly administrations of CFA (Fig. 1B). The discrepancy in pain-induced regulation of these two genes at later time points could be due to an array of factors, ranging from differences in susceptibility during long-term sensory regulation to diverse intracellular conditions required for induction of transcription or stabilization of the mRNAs. Besides having roles in activity-dependent excitability, BDNF is also important for neuronal survival, regeneration, outgrowth and overall maintenance [42], suggesting that continuous expression of the BDNF gene may be more important in development of latent stages of chronic pain. Overall, the initial nociception-induced up-regulation of NK-1 receptor and BDNF gene expression in the spinal cord supports the requirement of these neuromodulators for the maximum amplification of ascending pain signaling during central sensitization.

Nociceptive information from the periphery and the spinal cord is transmitted to the brain mainly through spinothalamic and parabrachial ascending pathways [43]. Furthermore, complex neuronal networks connect the parabrachial area or the thalamus to the limbic regions such as the hippocampus, the amygdala and the hypothalamus, which can modulate spinal nociceptive processing through activation of descending monoaminergic pathways from the brain stem [44]. However, the limbic areas of the brain, particularly the hippocampus, are also involved in control of stress responses and are considered to be the main regulators of mood or affect. CFA evoked robust decreases in NK-1 receptor and BDNF mRNA levels in the hippocampal formation, contrary to the prominent up-regulation seen in the spinal cord. A similar type of hippocampal plasticity was previously observed after either peripheral formalin injections or immobilization stress, as both of these stimuli were shown to down-regulate hippocampal NK-1 and BDNF gene expression [36,38,41]. The mechanisms responsible for the similarity between nociception- and stress-evoked effects in the hippocampus are not fully understood, but previous results suggest that maintenance of the nociceptive regulation of the hippocampus is not dependent upon continuous activation of the HPA axis [41]. The current results provide additional evidence that persistent nociception may have a significant effect on modulation of the higher brain centers, further suggesting that both the NK-1 receptor and BDNF may play a prominent role in pain processing within limbic structures such as the hippocampus.

Previous studies have suggested that CNS events leading to central sensitization during peripheral inflammation, hippocampal regulation due to stress, or long-term potentiation associated with spatial learning and memory may be dependent on similar intracellular transduction mechanisms, primarily the activation of cytosolic ERK and subsequent phosphorylation of nuclear CREB [29,45-50]. Both of these proteins are important for activity-dependent gene expression, and are thought to play a key role in synaptic plasticity contributing to translation of acute stimuli into long-term events such as the development and maintenance of chronic pain or depression. Western blot analysis showed that 24 h or 21 days after CFA administration, p-ERK levels were generally not altered in either the dorsal horn or the hippocampus, except for slight increases in spinal p-ERK2 (Fig. 4). Previous reports suggest that robust activation of spinal ERK following CFA [25,26,50] may be transient, since p-ERK levels usually peak within minutes after application of painful stimulus and then start to diminish due to increased dephosphorylation by various MAP kinase phosphatases [25,26,50]. We addressed initial p-ERK activation by assessing protein levels at 10 min after 50 μL of CFA (data not shown); however, changes in the regulation of either spinal or hippocampal p-ERK levels were not observed at this time point either. The discrepancy with previous reports may be due to a lesser amount of CFA used in our experiments, methods and/or quantitiative end-points used in each study, or differences in specific time points when protein changes were measured. However, eventual activation of transduction proteins such as ERKs and other intracellular kinases often initiates long-term synaptic plasticity through modulation of post-translational and transcriptional events that outlast both the initiating stimulus and kinase activation [25]. This may, in part, explain increased levels of nuclear p-CREB in the ipsilateral dorsal horn at both 24 h and 21 days (3×) after CFA treatments (Fig. 5). Elevated activation of CREB in the spinal cord further supports the role of this transcription factor in persistent nociception. Similar to NK-1 receptor and BDNF gene expression, CFA diminished hippocampal p-CREB levels, a phenomenon reminiscent of that previously reported during stress and depression [24,51], possibly by triggering intracellular events common to both types of stimuli. Besides ERKs, the activation of CREB may be coupled to other transduction factors, primarily the calcium/calmodulin-dependent protein kinase (CaM kinases)- or protein kinase A (PKA)-dependent cascades [27,52], to trigger the transcription of target genes such as NK-1 receptor and BDNF. Due to their similar patterns of nociception-evoked regulation, these data imply that expression of NK-1 receptor and BDNF genes could be at least partially controlled by CREB-dependent intracellular signaling in both the sensory and affective systems of the CNS [32]. However, the transcription of NK-1 receptor gene at the later phase of inflammation is probably dependent on other regulatory pathways, since no changes were observed in the dorsal horn 21 days after CFA.

## Conclusion

Our original hypothesis was that NK-1 receptor and BDNF gene expression may be co-regulated by peripheral nociception, but the results of this study demonstrated that is not the case, particularly at the longest time points tested in our chronic pain model. However, diverse patterns of gene expression suggest that NK-1 receptors and BDNF may have different roles in various regions of the CNS during chronic pain, since the spinal cord is an integral component of the sensory system, while the hippocampus is a part of the limbic system and a key contributor in regulation of affect. Novel information about the modulation of sensitivity, function, and plasticity of the genes encoding relevant neurotransmitters and their receptors, which contribute to the overall perception of pain, may give us a better idea of how to control the negative effects of pain on the mood and may provide a major insight into the development of improved therapeutic regimens for treating depression-like aspects of chronic pain.

## Abbreviations

ANOVA = analysis of variance

BDNF = brain-derived neurotrophic factor

CaM kinase = calcium/calmodulin-dependent protein kinase

cAMP = cyclic adenosine 3', 5' – monophosphate

CFA = complete Freund's adjuvant

CNS = central nervous system

CRE = cAMP response element

CREB = cAMP response element binding protein

ERK = extracellular signal-regulated kinase

MAPK = mitogen-activated protein kinase

NK-1 = neurokinin-1

PKA = protein kinase A

SP = substance P

TrkB = tyrosine kinase B

## Competing interests

The author(s) declare that they have no competing interests.

## Authors' contributions

VD and KMcC participated equally in the conception, design, data analysis and interpretation of the study. Additionally, VD carried out the animal handling and molecular analyses. All authors read and approved the final manuscript.
